# Mocapy++ - A toolkit for inference and learning in dynamic Bayesian networks

**DOI:** 10.1186/1471-2105-11-126

**Published:** 2010-03-12

**Authors:** Martin Paluszewski, Thomas Hamelryck

**Affiliations:** 1 Bioinformatics Centre, University of Copenhagen, Denmark

## Abstract

**Background:**

Mocapy++ is a toolkit for parameter learning and inference in *dynamic Bayesian networks *(DBNs). It supports a wide range of DBN architectures and probability distributions, including distributions from directional statistics (the statistics of angles, directions and orientations).

**Results:**

The program package is freely available under the *GNU General Public Licence *(GPL) from SourceForge http://sourceforge.net/projects/mocapy. The package contains the source for building the Mocapy++ library, several usage examples and the user manual.

**Conclusions:**

Mocapy++ is especially suitable for constructing probabilistic models of biomolecular structure, due to its support for directional statistics. In particular, it supports the Kent distribution on the sphere and the bivariate von Mises distribution on the torus. These distributions have proven useful to formulate probabilistic models of protein and RNA structure in atomic detail.

## Background

A *Bayesian network *(BN) represents a set of variables and their joint probability distribution using a directed acyclic graph [1,2]. A *dynamic Bayesian network *(DBN) is a BN that represents sequences, such as time-series from speech data or biological sequences [3]. One of the simplest examples of a DBN is the well known *hidden Markov model *(HMM) [4,5]. DBNs have been applied with great success to a large number of problems in various fields. In bioinformatics, DBNs are especially relevant because of the sequential nature of biological molecules, and have therefore proven suitable for tackling a large number of problems. Examples are protein homologue detection [6], protein secondary structure prediction [7,8], gene finding [5], multiple sequence alignment [5] and sampling of protein conformations [9,10].

Here, we present a general, open source toolkit, called Mocapy++, for inference and learning in BNs and especially DBNs. The main purpose of Mocapy++ is to allow the user to concentrate on the probabilistic model itself, without having to implement customized algorithms. The name Mocapy stands for *Markov chain ****Mo****nte ****Ca****rlo and ****Py****thon*: the key ingredients in the original implementation of Mocapy (T. Hamelryck, University of Copenhagen, 2004, unpublished). Today, Mocapy has been re-implemented in C++ but the name is kept for historical reasons. Mocapy supports a large range of architectures and probability distributions, and has proven its value in several published applications [9-13]. This article serves as the main single reference for both Mocapy and Mocapy++.

### Existing Packages

Kevin Murphy maintains a list of software packages for inference in BNs [14]. Currently, this list contains 54 packages. A small subset of these packages share some key features with Mocapy++ (see Table 1). These packages have an *application programming interface *(API), perform parameter estimation and are free of charge (at least for academic use). Mocapy++ is mainly intended for use in scientific research, where reproducibility and openness of scientific results are important. Commercial closed source packages are therefore not included in this discussion.

**Table 1:** Some popular Free BN packages with an API. Extracted from Murphy [14].

Name	Authors	Source	Inference	Learning
Bayes Blocks	Harva et al. [30]	Python/C++	Ensemble learning	VB
BNT	Murphy [31]	Matlab/C	JTI, MCMC	EM
BUGS	Lunn et al. [32]	N	Gibbs	Gibbs
Elvira	Elvira Consortium [33]	Java	JTI, IS	EM
Genie	U. Pittsburgh [34]	N	JTI	EM
GMTk	Blimes, Zweig [35]	N	JTI	EM
Infer.NET	Winn and Minka [36]	C#	BP, EP, Gibbs, VB	EP
JAGS	Plummer	C++	Gibbs	Gibbs
Mocapy++	Paluszewski and Hamelryck	C++	Gibbs	S-EM, MC-EM

The abbreviations are N: source code is not freely available, BP: belief propagation, EP: expectation propagation, JTI: junction tree inference, Gibbs: Gibbs sampling, MCMC: Markov chain Monte Carlo, VB: variational Bayes, IS: importance sampling. JAGS is available online from http://www-fis.iarc.fr/~martyn/software/jags/

In bioinformatics, models are typically trained using large datasets. Some packages in Table 1 only provide exact inference algorithms that are often not suitable for training models with large datasets. Other packages have no or little support for DBNs, which is important for modelling biomolecular structure. To our knowledge none of the publically available open source toolkits support directional statistics, which has recently become of crucial importance for applications in structural bioinformatics such as modelling protein and RNA structure in 3D detail [9,10,12,15]. Furthermore, Mocapy++ is the only package that uses the stochastic EM [16-18] algorithm for parameter learning (see the Materials and Methods section). These features make Mocapy++ an excellent choice for many tasks in bioinformatics and especially structural bioinformatics.

## Implementation

Mocapy++ is implemented as a program library in C++. The library is highly modular and new node types can be added easily. For object serialization and special functions the Boost C++ library [19] is used. All relevant objects are serializable, meaning that Mocapy++ can be suspended and later resumed at any state during training or sampling. The LAPACK library [20] is used for linear algebra routines.

Mocapy++ uses CMake [21] to locate packages and configure the build system and can be used either as a static or shared library. The package includes a Doxygen configuration file for HTML formatted documentation of the source code. An example of a Python interface file for SWIG http://www.swig.org is also included in the package.

### Data structures

Most of the internal data is stored in simple *Standard Template Library *(STL) [22] data structures. However, STL or other public libraries offer little support for multidimensional arrays when the dimension needs to be set at run-time. In Mocapy++ such a multidimensional array is for example needed to store the *conditional probability table *(CPT) of the discrete nodes. The CPT is a matrix that holds the probabilities of each combination of node and parent values. For example, a discrete node of size 2 with two parents of sizes 3 and 4, respectively, will have a 3 × 4 × 2 matrix as its CPT. Mocapy++ therefore has its own implementation of a multidimensional array, called MDArray. The MDArray class features dynamic allocation of dimensions and provides various slicing operations. The MDArray is also used for storing the training data and other internal data.

### Specifying a DBN in Mocapy++

**Figure 1:**
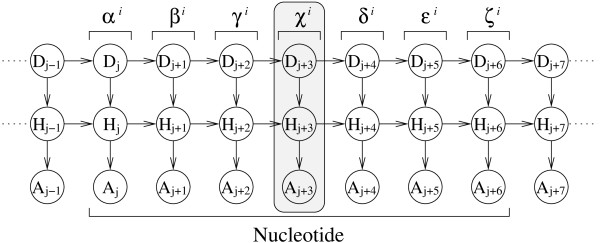
**BARNACLE: a probabilistic model of RNA structure**. A DBN with nine slices is shown, of which one slice is boxed. Nodes *D *and *H *are discrete nodes, while node *A *is a univariate von Mises node. The dihedral angles within one nucleotide *i *are labelled *α*^*i *^to *ζ*^*i*^. BARNACLE is a probabilistic model of the dihedral angles in a stretch of RNA [12].

Consider a sequence of observations. Each position in the sequence is characterized by *n *random variables (called a *slice*, see Figure 1). Each slice in the sequence can be represented by an ordinary BN, which is duplicated along the sequence as necessary. The sequential dependencies are in turn represented by edges between the consecutive slices. Hence, a DBN in Mocapy++ is defined by three components: a set of nodes that represent all variables for a given slice, the edges between the nodes within a slice (the *intra edges*) and the edges that connect nodes in two consecutive slices (the *inter edges*).

### Node Types

Mocapy++ supports several node types, each corresponding to a specific probability distribution. The categorical distribution (discrete node), multinomial (for vectors of counts), Gaussian (uni- and multivariate), von Mises (uni- and bivariate; for data on the circle or the torus, respectively) [23], Kent (5-parameter Fisher-Bingham; for data on the sphere) [24] and Poisson distributions are supported. Some node types, such as the bivariate von Mises and Kent nodes, are to our knowledge only available in Mocapy++. The bivariate von Mises and Kent distributions are briefly described here. These distributions belong to the realm of directional statistics, which is concerned with probability distributions on manifolds such as the circle, the sphere or the torus [23,25].

#### Kent Distribution

The Kent distribution [9,24,26-29], also known as the 5-parameter Fisher-Bingham distribution, is a distribution on the 2D sphere (the surface of the 3D ball). It is the 2D member of a larger class of *N*-dimensional distributions called the Fisher-Bingham distributions. The density function of the Kent distribution is:

where **x **is a random 3D unit vector that specifies a point on the 2D sphere.

The various parameters can be interpreted as follows:

• *κ*: a concentration parameter. The concentration of the density increases with *κ*.

• *β*: determines the ellipticity of the equal probability contours of the distribution. The ellipticity increases with *β*. If *β *= 0, the Kent distribution becomes the von Mises-Fisher distribution on the 2D sphere.

• *γ*_1_: the mean direction.

• *γ*_2_: the main axis of the elliptical equal probability contours.

• *γ*_3_: the secondary axis of the elliptical equal probability contours.

The normalizing factor *C*(*κ*, *β*) is approximately given by:

The Kent distribution can be fully characterized by 5 independent parameters. The concentration and the shape of the equal probability contours are characterized by the *κ *and *β *parameters, respectively. Two angles are sufficient to specify the mean direction on the sphere, and one additional angle fixes the orientation of the elliptical equal probability contours. The latter three angles are in practice specified by the three orthonormal *γ *vectors, which form a 3 × 3 orthogonal matrix.

**Figure 2:**
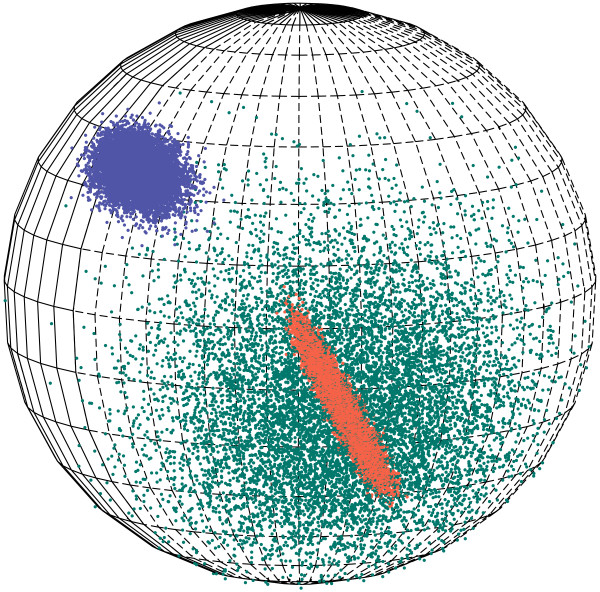
**Samples from three Kent distributions on the sphere**. The red points were sampled from a distribution with high concentration and high correlation (*κ *= 1000, *β *= 499), the green points were sampled from a distribution with low concentration and no correlation (*κ *= 10, *β *= 0), and the blue points were sampled from a distribution with medium concentration and medium correlation (*κ *= 200, *β *= 50). The distributions underlying the red and green points have the same mean direction and axes and illustrate the effect of *κ *and *β*. For each distribution, 5000 points are sampled.

The advantage of the Kent distribution over the von Mises-Fisher distribution on the 2D sphere is that the equal probability contours of the density are not restricted to be circular: they can be elliptical as well. The Kent distribution is equivalent to a Gaussian distribution with unrestricted covariance. Hence, for 2D directional data the Kent distribution is richer than the corresponding von Mises-Fisher distribution, i.e. it is more suited if the data contains non-circular clusters. The Kent distribution is illustrated in Figure 2. This distribution was used to formulate FB5HMM [9], which is a probabilistic model of the local structure of proteins in terms of the *Cα *positions.

#### Bivariate von Mises Distribution

Another distribution from directional statistics is the bivariate von Mises distribution on the torus [23]. This distribution can be used to model bivariate angular data. The density function of the bivariate von Mises (cosine variant) distribution is:

where *C*(*κ*_1_, *κ*_2_, *κ*_3_) is the normalizing factor and *ϕ*, *ψ *are random angles in [0, 2*π*[. Such an angle pair defines a point on the torus.

The distribution has 5 parameters:

• *μ *and *ν *are the means for *ϕ *and *ψ *respectively.

• *κ*_1 _and *κ*_2 _are the concentration of *ϕ *and *ψ *respectively.

• *κ*_3 _is related to their correlation.

**Figure 3:**
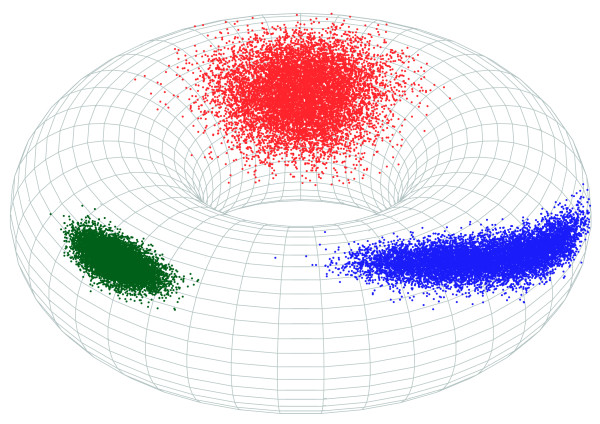
**Samples from three bivariate von Mises distributions on the torus**. The green points were sampled from a distribution with high concentration and no correlation (*κ*_1 _= *κ*_2 _= 100, *κ*_3 _= 0), the blue points were sampled from a distribution with high concentration and negative correlation (*κ*_1 _= *κ*_2 _= 100, *κ*_3 _= 49), and the red points were sampled from a distribution with low concentration and no correlation (*κ*_1 _= *κ*_2 _= 10, *κ*_3 _= 0). For each distribution, 10000 points are sampled.

This distribution is illustrated in Figure 3 and described in greater detail in Mardia *et al. *[23]. The distribution was used by Boomsma *et al. *[10] to formulate a probabilistic model of the local structure of proteins in atomic detail.

### Inference and Learning

Mocapy++ uses a *Markov chain Monte Carlo *(MCMC) technique called Gibbs sampling [1] to perform inference, i.e. to approximate the probability distribution over the values of the hidden nodes. Sampling methods such as Gibbs sampling are attractive because they allow complicated network architectures and a wide range of probability distributions.

Parameter learning of a DBN with hidden nodes is done using the *expectation maximization *(EM) method, which provides a maximum likelihood point estimate of the parameters. In the E-step, the values of the hidden nodes are inferred using the current DBN parameters. In the subsequent M-step, the inferred values of the hidden nodes are used to update the parameters of the DBN using maximum likelihood estimation. The E- and M-step cycle is repeated until convergence. Parameter learning using the EM algorithm requires a method to perform inference over the possible hidden node values. If one uses a stochastic procedure to perform the E-step (as in Mocapy++), a stochastic version of the EM algorithm is obtained. There are two reasons to use a stochastic E-step. First, deterministic inference might be intractable. Second, certain stochastic versions of the EM algorithm are more robust than the classic version of EM [16]. EM algorithms with a stochastic E-step come in two flavors [1,17]. In *Monte Carlo EM *(MC-EM), a large number of samples is generated in the EM step. In *Stochastic EM *(S-EM) [16-18] only one sample is generated for each hidden node, and a 'completed' dataset is obtained. In contrast to MC-EM, S-EM has some clear advantages over deterministic EM algorithms: S-EM is less dependent on starting conditions, and has a lower tendency to get stuck at saddle points, or insignificant local maxima. Because only one value needs to be sampled for each hidden node in the E-step, S-EM can also be considerably faster than MC-EM. S-EM is especially suited for large datasets, while for small datasets MC-EM is a better choice. Mocapy++ supports both forms of EM.

## Results and Discussion

Hamelryck *et al. *[9] sample realistic protein *Cα*-traces using an HMM with a Kent output node. Boomsma *et al. *[10] extend this model to full atomic detail using the bivariate von Mises distribution [23]. In both applications, Mocapy was used for parameter estimation and sampling. Zhao *et al. *[11] used Mocapy for related work. Mocapy has also been used to formulate a probabilistic model of RNA structure [12] (Figure 1) and to infer functional interactions in a biomolecular network [13].

**Figure 4:**
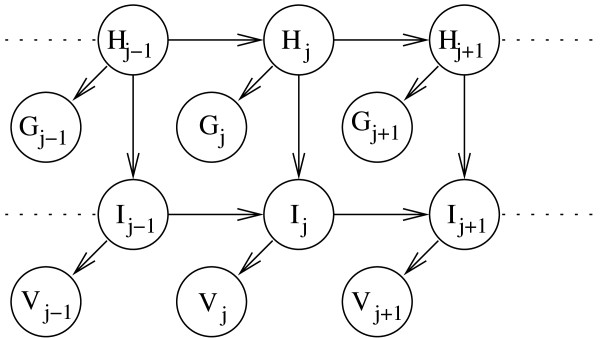
**The model used in the third benchmark**. Each slice contains two hidden nodes (*H *and *I*). They are parents to a multivariate four-dimensional Gaussian node (*G*) and a bivariate von Mises node (*V*), respectively. The sizes of *H *and *I *are five and three, respectively. The length of the BN is 50 slices.

To illustrate the speed of Mocapy++, we use three parameter estimation benchmarks and report the execution time on a standard PC (Intel Core 2 Duo, 2.33 GHz). The first benchmark is an HMM with 50 slices and two discrete nodes in each slice (one hidden node and one output node). All nodes have 5 states. The second benchmark is similar, but with a 4-dimensional Gaussian output node and a 10 state hidden node. The third benchmark is more complex and is shown in Figure 4.

**Figure 5:**
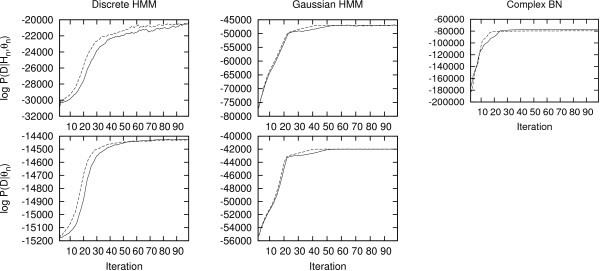
**Log-likelihood evolution during S-EM training**. Each column shows the evolution of the log-likelihood for one of the three benchmarks described in the results section. The training procedure was started from two different random seeds (indicated by a solid and a dashed line). The log-likelihood values, log *P *(*D*|*H*_*n*_, *θ*_*n*_), used in the upper figures are conditional on the states of the sampled hidden nodes (*θ*_*n *_are the parameter values at iteration *n*, *H*_*n *_are the hidden node values at iteration *n *and *D *is the observed data). The log-likelihood values in the lower figures, log *P *(*D*|*θ*_*n*_), are computed by summing over all hidden node sequences using the forward algorithm [5]. Note that the forward algorithm can only be used on HMMs and is therefore not applied on the complex benchmark.

Using a training set consisting of 200 sequences, 100 iterations of S-EM take 14 seconds for the discrete HMM, 41 seconds for the Gaussian HMM and 195 seconds for the complex BN. The evolution of the log-likelihood during training is shown in Figure 5.

In practice, the most time consuming step in parameter learning is Gibbs sampling of the hidden nodes. The running time for one sweep of Gibbs sampling for a hidden discrete node is *O*(*l *× *s*) where *l *is the total number of slices in the data and *s *is the size of the node. The largest model that, to our knowledge, has been successfully trained with Mocapy++ is an extension of TorusDBN [10]. The dataset consisted of 9059 sequences with a total of more than 1.5 million slices. The model has 11897 parameters and one EM-iteration takes 860 seconds. The number of S-EM iterations needed for likelihood convergence is around 100.

Toolkits for inference and learning in Bayesian networks use many different algorithms and are implemented in a variety of computer languages (Matlab, R, Java,...); comparisons are thus necessarily unfair or even irrelevant. Therefore, we feel it suffices to point out that Mocapy++ has some unique features (such as the support for directional statistics), and that the benchmarks clearly show that its performance is more than satisfactory for real life problems - both with respect to speed and data set size.

### Future Directions of Mocapy++

The core of Mocapy++ described here is not expected to change much in future versions of Mocapy++. However, Mocapy++ is an evolving project with room for new features and additions. We therefore encourage people to propose their ideas for improvements and to participate in the development of Mocapy++. Potential directions include:

• Additional probability distributions

• Structure learning

• Graphical user interface

• Plugins for reading data in various formats

## Conclusions

Mocapy++ has a number of attractive features that are not found together in other toolkits [14]: it is open source, implemented in C++ for optimal speed efficiency and supports directional statistics. This branch of statistics deals with data on unusual manifolds such as the sphere or the torus [25], which is particularly useful to formulate probabilistic models of biomolecular structure in atomic detail [9-12]. Finally, the use of S-EM for parameter estimation avoids problems with convergence [16,17] and allows for the use of large datasets, which are particularly common in bioinformatics. In conclusion, Mocapy++ provides a powerful machine learning tool to tackle a large range of problems in bioinformatics.

## Availability and Requirements

• Project name: Mocapy++

• Project home page: http://sourceforge.net/projects/mocapy

• Operating system(s): Linux, Unix, Mac OS X, Windows with Cygwin

• Programming language: C++

• Other requirements: Boost, CMake and LAPACK, GNU Fortran

• License: GNU GPL

## Competing interests

The authors declare that they have no competing interests.

## Authors' contributions

TH designed and implemented Mocapy in Python. MP designed and implemented Mocapy++. MP drafted the manuscript and TH revised the manuscript. Both authors read and approved the final manuscript.
